# The time has come to protect healthcare workers and patients from aerosol transmissible disease

**DOI:** 10.3389/fpubh.2024.1378567

**Published:** 2024-04-23

**Authors:** Lisa M. Brosseau, Andrew Bowdle, Raymond Tellier, Michael Klompas, Robert T. Schooley, Robert Harrison, Srdjan Jelacic, Michael T. Osterholm

**Affiliations:** ^1^Center for Infectious Disease Research and Policy, University of Minnesota, Minneapolis, MN, United States; ^2^Department of Anesthesiology, University of Washington, Seattle, WA, United States; ^3^Department of Medicine, McGill University, Montreal, QC, Canada; ^4^Departments Medicine and Population Medicine, Harvard Medical School, Boston, MA, United States; ^5^Department of Medicine, University of California San Diego, San Diego, CA, United States; ^6^Department of Medicine, University of California San Francisco, San Francisco, CA, United States

**Keywords:** respirator, pandemic, SARS-CoV-2, respiratory protection, mask, aerosol transmission, indoor air, N95

## Introduction

In order to protect patients and healthcare workers from aerosol transmissible diseases, healthcare facilities should improve ventilation and air purification and in addition should consider universal use of respirators (e.g., N95, FFP2 or equivalent) when aerosol-transmissible pathogens are widespread in the community. A study of SARS-CoV-2 within 288 United States hospitals documented more than 14,000 infections potentially acquired in the hospital over a 2-year period and found that more than 8% of patients hospitalized with SARS-CoV-2 may have acquired their infection in the hospital (1). Despite the frequency of nosocomial respiratory viral transmission most countries have no national mandate for masks or respirators in healthcare facilities. We propose that healthcare facilities should anticipate that aerosol transmissible disease will continue to be of major importance to public health for the foreseeable future.

## Aerosol transmissible disease

Early in the COVID pandemic, some argued that SARS CoV-2 was transmitted primarily by larger respiratory particles known as “droplets,” produced during coughing and sneezing and propelled into the mouth or nose of someone nearby (2). Surgical masks were thought by many to provide adequate protection; respirators were only recommended for healthcare workers performing a limited and variable set of procedures.

Examination of a large body of evidence has shown that transmission of SARS-CoV-2 is primarily through inhalation of smaller respiratory particles generated by breathing, talking, singing and other ordinary respiratory activities (3–7). These smaller particles are predominantly < 5 microns in size, can remain suspended in air for many minutes or even hours, diffuse or move by air currents throughout an indoor space, and are easily inhaled both near and far from a source (8). Optimal protection from inhaling these small particles requires respirators, not surgical masks (4, 9). While recent attention has been focused on SARS-CoV-2, the importance of aerosol transmission for a wide variety of pathogens has been recognized for many years; examples of aerosol transmissible viruses and bacteria are shown in the Table 1, along with examples of pertinent literature.

**Table 1:** Examples of aerosol transmissible pathogens.

**Pathogen**	**Early evidence of aerosol transmission**	**Person to person transmission**
Adenovirus	Couch et al. (10)	Yes
Coxiella burnetti (Q fever)^*^	Welsh et al. (11)	No
Coxsackie A21 virus	Couch et al. (12)	Yes
Influenza virus	Alford et al. (13)	Yes
Legionella pneumophila	Nguyen et al. (14)	No
Mycobacterium tuberculosis	Riley et al. (15)	Yes
Respiratory syncytial virus	Kulkarni et al. (16)	Yes
Rubella virus (measles)	Marks et al. (17)	Yes
Rubeola virus (measles)	Riley et al. (18)	Yes
SARS-CoV-2 virus (COVID)	Hamner et al. (19)	Yes
Staphylococcus aureus	Eichenwald et al. (20)	Yes
Varicella virus (chicken pox)	Leclair et al. (21)	Yes
Variola virus (smallpox)^*^	Wehrle et al. (22)	Yes
Yersinia pestis (pneumonic plague)^*^	Meyer (23)	Yes

^*^Potential bioweapon.

Examples of pathogens with significant aerosol transmission, along with a single representative citation for each. This list of pathogens and citations is not intended to be inclusive or exhaustive. The citations were selected to emphasize that evidence for aerosol transmission of a number of pathogens has been available for more than 60 years.

## Variable performance of face coverings

Recognition of the importance of aerosol transmission has critical implications for healthcare workers and patients. However, the conversation about appropriate protection has been obscured by the widespread use of the terms “mask” and “masking” to encompass anything worn on the face. These terms lack precision and suggest that everything worn on the face provides similar levels of source control and personal protection. In fact, there are large differences in the performance characteristics and effectiveness of different face coverings.

The most common face coverings in health care facilities are surgical masks (a term which encompasses procedure masks). Most surgical masks are not designed to fit tightly against the face and thus have a limited impact on inward and outward movement of smaller particles because air can move freely around the edges of the mask instead of through the filtering material. In addition, surgical masks are typically constructed of filtering material that is not as effective as the material used for respirators. Surgical masks have been worn during surgery with the intention of preventing bacterial infection of surgical wounds from droplets generated by surgical personnel, although efficacy for this purpose is questionable (24). Since the HIV epidemic, surgical masks have also been deployed as protection against splashes with blood or other body fluids. Efficacy of surgical masks for protection against aerosol transmission is limited (25–27).

Respirators (which should not be referred to as “masks”) are designed to fit tightly to the face, are constructed of highly effective filtering material and can provide substantial protection against aerosol transmission. They typically undergo a rigorous testing and approval process supervised by governmental agencies. The most common disposable “filtering facepiece” respirators are designated as N95 in the United States and Canada and FFP2 in the United Kingdom, European Union, Australia and New Zealand.

There are some in healthcare who question the relative effectiveness of respirators in comparison to surgical masks. Laboratory and workplace measurements have clearly and consistently demonstrated the superior performance of respirators for all types of hazardous aerosols (26–28). However, some have suggested that evidence from randomized clinical trials is needed. This is problematic because most trials have only assigned healthcare workers to wear respirators when caring for patients with known or suspected respiratory viral infections, ignoring the fact that workers are continuously exposed to viruses in other contexts, at home, in the community and from exposure to co-workers and pre-symptomatic or asymptomatic patients. Nevertheless, the limited clinical evidence we have suggests that respirators reduce the risk of infection to a greater degree than surgical masks (29–31), consistent with the strong evidence from laboratory and workplace measurements of respirator performance.

## Discussion

The HIV epidemic transformed healthcare worker behavior, making contact between the healthcare worker and patient body fluids something to be strictly avoided. Similarly, the COVID-19 pandemic has heightened awareness about the importance of preventing transmission of aerosolized pathogens (32). Prior to COVID-19, precautions against aerosol-transmissible pathogens were considered important for only a few specific pathogens, such as tuberculosis and measles and most healthcare workers seldom if ever donned a respirator. In fact, evidence for aerosol transmission of influenza has been accumulating since the 1960s (33) along with evidence for aerosol transmission of a variety of viruses (10, 34), bacteria (35) and fungi (36). Coronaviruses and influenza viruses are especially noteworthy because of their proven pandemic potential (37), but their mode of transmission is not unique. Some pathogens, not classically thought to spread by aerosols, can become airborne pathogens in some circumstances, for example *Yersinia pestis* in primary plague pneumonia (23, 38). While not all aerosol transmissible diseases result in a significant incidence of hospitalization and death, many are serious threats to public health.

The importance of aerosol transmission has fundamental importance for health policy, because traditional droplet precautions, such as staying six feet away from a source or wearing a surgical mask, will not provide adequate protection from aerosols. Prevention of aerosol transmission requires attention to indoor air quality through adequate ventilation and air purification and the use of respirators rather than surgical masks for personal protection and source control.

Since universal masking with either respirators or surgical masks has been largely abandoned by healthcare facilities, it is critical to understand the appropriate triggers for reinstating universal respiratory protection. This has been the subject of considerable discussion but unfortunately remains unclear (39). Knowing when to upgrade or relax precautions depends upon reliable and timely assessment of transmission and the consequences of infection. This is not a trivial problem. For instance, in many places around the world including some parts of the United States, testing and reporting of COVID-19 infection, hospitalization and death has lapsed, and wastewater monitoring has become the main source of data used to infer prevalence.

We need to develop better ways to monitor our environment for indicators of respiratory pathogen risk in near real time and geolocatable terms, and to use this information in quantitative ways to assess respiratory risk. For example, Puthussery et al. (40) recently reported a technology for near real-time analysis of air samples for SARS-CoV-2 or other viruses that might be used to estimate the risk of transmission from indoor air for a specific time and location. Similar technologies might also be used to perform near real-time testing of exhaled air from individuals to identify infection and the need for source control (41). While SARS-CoV-2 has been of greatest concern in recent times, the risks posed by other aerosol transmissible pathogens, or the combined risks from several pathogens circulating simultaneously in a community may warrant elevated precautionary measures; such protocols must become part of preparedness for future pandemics, some of which will involve, no doubt, airborne pathogens.

Lacking methods for sampling infectious aerosol concentrations in indoor spaces, it may be possible to assess risk qualitatively. Important factors include indoor air quality, based on ventilation and air purification, the likely number of encounters with potential sources (while noting that many infected persons can be asymptomatic or presymptomatic) and duration of exposure. When community transmission of a respiratory pathogen is widespread, exposure to healthcare workers and visitors with occult infections, in addition to infected patients, becomes a risk factor.

Universal masking policies in healthcare facilities have to consider potential obstacles to compliance including the available supply of masks and respirators. In some countries, regulators require periodic fit testing of respirators for employees who may be required to use them in the workplace. While the use of fit tested respirators is more likely to provide optimal protection from aerosol transmission than when respirators are worn without fit testing, a well-designed respirator that fits most people well is likely to provide better protection than surgical masks or other relatively less effective face coverings (26). Thus, providing respirators for use by patients, even when not fit tested, may be a rational protective measure. Whether healthcare workers or patients can or should be compelled to use respirators, surgical masks, or other face coverings is a complex legal, political and administrative problem with no easy answers.

Some have expressed practical concerns about the supply of respirators. At the current time, respirators are in abundant supply, however early in the COVID-19 pandemic this was not the case. When respirators are in short supply, it is important to realize that a single filtering facepiece respirator (e.g., N95, FFP2) can be worn at least a few times without losing its ability to fit and filter effectively. Once trapped in the filtering material of the respirator, particles remain bound indefinitely (42, 43). Greater routine use of respirators might well stimulate improvements in design that improve comfort and ease of donning and doffing; and a steady demand for respirators might make the supply chain more robust. Reusable elastomeric respirators offer advantages in comparison to disposable respirators (44) because they can be cleaned and their filters are very long lasting. Especially in pandemic or surge situations, elastomeric respirators have considerable strategic value.

In conclusion, there is convincing evidence for aerosol transmission of many pathogens, including some with pandemic potential, such as influenza and corona viruses. Healthcare facilities should endeavor to improve ventilation and air purification to reduce exposure of healthcare workers and patients to dangerous aerosols. When the risk of aerosol transmission is elevated, especially when transmission in the community is widespread, masking healthcare workers and patients, preferably with respirators rather than surgical masks, will make healthcare safer for all.

## Author contributions

LB: Conceptualization, Writing – original draft, Writing – review & editing. AB: Conceptualization, Writing – original draft, Writing – review & editing. RT: Writing – review & editing. MK: Writing – review & editing. RS: Writing – review & editing. RH: Writing – review & editing. SJ: Writing – review & editing. MO: Writing – review & editing.
